# Association between urinary NNAL concentrations and osteoarthritis: Evidence from NHANES 2007–2012

**DOI:** 10.18332/tid/236205

**Published:** 2026-09-23

**Authors:** Jun Liu, Jihong Zhang, Zhou Guo, Zhipeng Wang, Song Zhou, Junming Huang

**Affiliations:** 1 The Orthopedic Hospital, The First Affiliated Hospital, Jiangxi Medical College, Nanchang University, Nanchang, China; 2 Department of Sports Medicine, Orthopedic Hospital, The First Affiliated Hospital, Jiangxi Medical College, Nanchang University, Nanchang, China; 3 The Key Laboratory of Spine and Spinal Cord Diseases of Jiangxi Province, Nanchang, China; 4 Department of Pain Medicine, The First Affiliated Hospital, Jiangxi Medical College, Nanchang University, Nanchang, China; 5 Postdoctoral Research Station, The First Affiliated Hospital, Jiangxi Medical College, Nanchang University, Nanchang, China

**Keywords:** osteoarthritis, smoking, NNAL, epidemiology

## Abstract

**INTRODUCTION:**

Osteoarthritis (OA) is the most prevalent degenerative joint disorder globally, yet the impact of smoking on its development remains contentious. The compound 4-(methylnitrosamino)-1-(3-pyridyl)-1-butanol (NNAL), a principal metabolite of the tobacco-specific nitrosamine 4-(methylnitrosamino)-1-(3-pyridyl)-1-butanone (NNK), is a well-recognized biomarker for smoking exposure. This study aimed to use urinary NNAL as a biomarker to evaluate the association between tobacco exposure and OA.

**METHODS:**

This study was a secondary cross-sectional analysis of pooled data from the 2007–2012 cycles of the National Health and Nutrition Examination Survey (NHANES), including 10848 adults. OA was assessed using self-reported physician diagnosis and radiographic examination, with a Kellgren-Lawrence grade ≥2 indicating radiographic OA; urinary NNAL concentration was used as the biomarker of tobacco exposure. Survey-weighted multivariable logistic regression analysis and subgroup analyses were performed after adjusting for confounding factors.

**RESULTS:**

Elevated urinary NNAL concentrations were positively associated with greater odds of OA. In the fully adjusted model, urinary NNAL concentrations >0.077925 ng/mL were associated with greater odds of OA (adjusted odds ratio, AOR=1.55; 95% CI: 1.36–1.76). Evidence of interaction was observed for age (p for interaction <0.001), but not for sex (p=0.231) or BMI (p=0.127). Among adults aged <60 years, the AOR for the highest versus lowest NNAL category was 1.69 (95% CI: 1.46–1.95), whereas the corresponding AOR was 0.98 (95% CI: 0.82–1.17) among adults aged ≥60 years.

**CONCLUSIONS:**

Higher urinary NNAL was associated with an increased likelihood of OA, with more prominent effects observed in middle-aged adults. Given the cross-sectional design, temporality and causality cannot be established, and the potential association requires confirmation in longitudinal and mechanistic studies.

## INTRODUCTION

Osteoarthritis (OA) represents the most prevalent form of arthritis and constitutes a leading cause of disability among adults on a global scale^1^. This degenerative joint disease is characterized by the progressive deterioration of articular cartilage, alterations in subchondral bone, and inflammation of the synovial membrane. According to the World Health Organization (WHO), approximately 595 million individuals worldwide are afflicted with OA, and this figure is projected to increase by 2050, positioning OA as one of the most significant chronic diseases affecting the elderly population^1^. Historically, the etiology of OA has been interpreted primarily through intrinsic factors, such as aging, obesity, and previous joint injuries^2,3^. Nevertheless, recent research has started to reveal the significant impact of extrinsic factors, particularly environmental pollutants, on the development and progression of OA^4^.

Among the myriad environmental factors, smoking has emerged as a significant focus of research due to its widespread prevalence and extensively documented health implications^5^. Traditionally linked to respiratory diseases and various cancers, smoking has also been implicated in the progression of numerous systemic disorders. Previous research has produced conflicting evidence regarding the relationship between smoking and OA^6^. Some studies suggest that smoking may exert a protective effect against OA. For instance, a cross-sectional study indicated a negative correlation between smoking and the prevalence of knee OA among the older Korean population^7^. Furthermore, a comprehensive review and meta-analysis of 34 independent observational studies published in 2017 demonstrated that cigarette smoking was inversely associated with knee OA, with a stronger association observed in males^8^. However, more recent investigations have indicated that smoking may adversely affect joint health and contribute to the onset and progression of OA. Evidence suggests that smokers may have a greater occurrence of OA-related symptoms and experience structural changes in their joints compared to non-smokers^9^. The divergent conclusions of these studies may be attributable to variations in the criteria used to assess smoking exposure.

Tobacco-specific nitrosamines (TSNAs) represent a class of toxic compounds that are distinct from other carcinogens present in cigarettes, such as heavy metals, due to their specificity to tobacco^10^. These substances are generated in tobacco through the nitrosation of nicotine alkaloids during processes such as roasting and fermentation. Among these nitrosamines, 4-(methylnitrosamino)-1-(3-pyridyl)-1-butanone (NNK) is a significant component of both tobacco products and tobacco smoke. NNK is rapidly metabolized into its derivative, 4-(methylnitrosamino)-1-(3-pyridyl)-1-butanol, commonly known as NNAL^11^. The presence of NNAL in urine serves as a reliable indicator of tobacco exposure^12^. Previous studies indicate that tobacco-specific NNAL has a longer half-life than cotinine, making it a more effective biomarker for delayed tobacco exposure. Urinary NNAL is a well-established biomarker of exposure to NNK, a tobacco-specific nitrosamine with genotoxic and carcinogenic properties^13^. Epidemiological studies have used urinary NNAL concentrations as objective indicators of exposure to NNK and tobacco smoke when examining a range of health outcomes, including cardiovascular and oral health outcomes^14-16^. To our knowledge, no previous study has used urinary NNAL to examine the association between tobacco exposure and OA. In this study, a nationally representative sample derived from the National Health and Nutrition Examination Survey (NHANES) was used to examine the association between tobacco exposure and OA by employing urinary NNAL.

## METHODS

This study was a secondary analysis of pooled data from the 2007–2012 cross-sectional cycles of the National Health and Nutrition Examination Survey (NHANES), a nationally representative survey of the non-institutionalized US population. NHANES systematically links clinical measurements, nutritional biomarkers, and social behavior determinants of health. The survey triangulates self-reported information with objective biophysical data, and every data-collection protocol begins only after participants provide written informed consent in full compliance with research ethics standards.

### Study population and inclusion criteria

**Figure 1:**
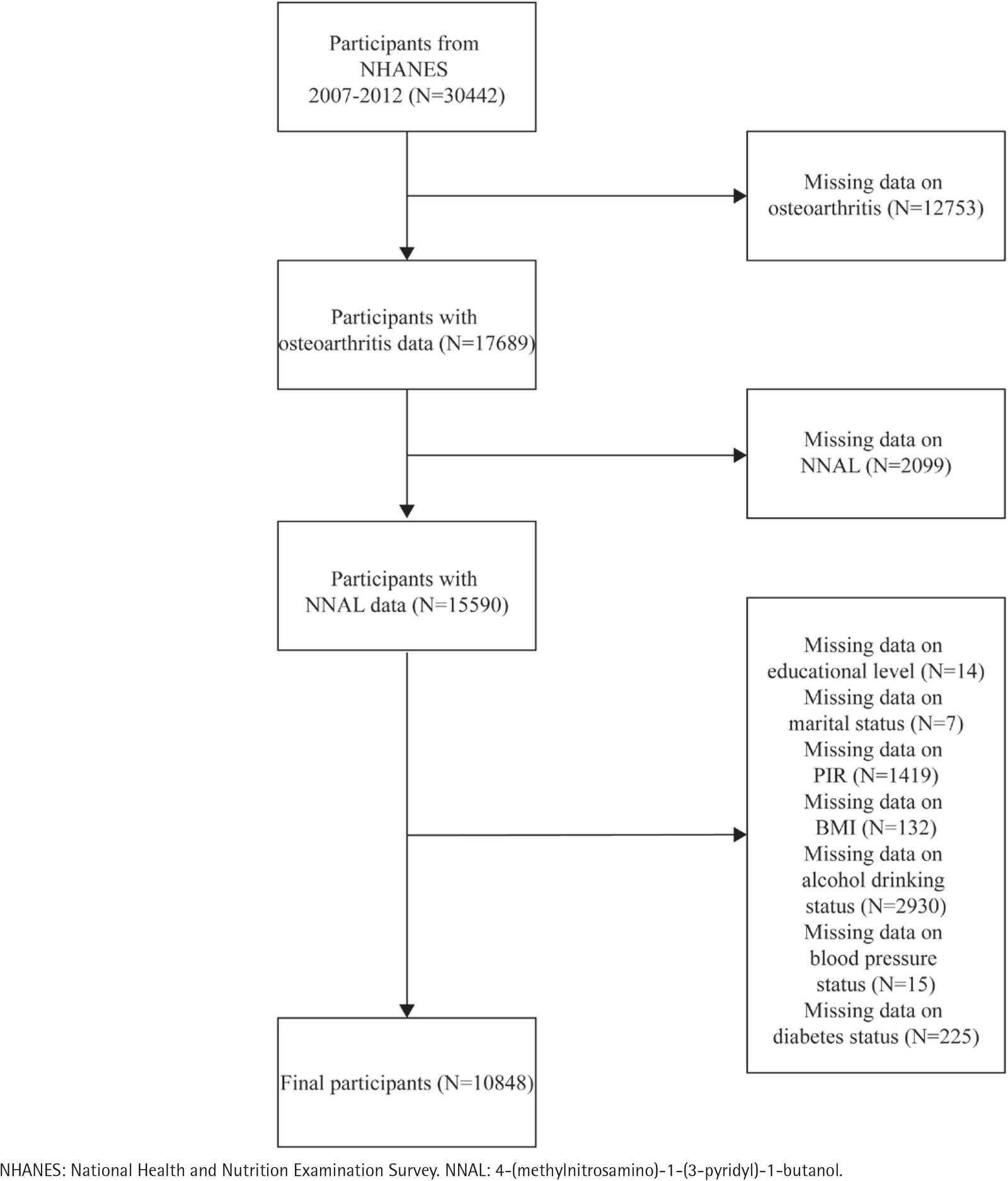
Participant selection flowchart for the analysis of the association between urinary NNAL concentrations and osteoarthritis, using pooled NHANES data, United States, 2007–2012

Among 30442 participants in the pooled 2007–2012 NHANES cycles, individuals were included if they had available data on OA status, urinary total NNAL concentrations, and all covariates used in the analysis. Participants with missing OA, urinary NNAL, or covariate data were excluded. The final analytic sample comprised 10848 participants. Detailed participant exclusions are presented in Figure 1.

### Assessment of osteoarthritis

At the mobile examination center, trained interviewers first ascertained physician-diagnosed osteoarthritis by asking participants whether a healthcare professional had ever explicitly told them they had ‘osteoarthritis’. Subsequently, certified technicians obtained bilateral knee and hip radiographs that were independently graded by two musculoskeletal radiologists who were blinded to clinical data; Kellgren–Lawrence (KL) grade ≥2 in any joint was considered radiographic osteoarthritis^17^.

### NNAL in urine

The tobacco-specific nitrosamine 4-methylnitrosamino-1-3-pyridyl-1-butanone (NNK) is a major constituent of tobacco and tobacco smoke. In humans, NNK is rapidly reduced to its metabolite 4-methylnitrosamino-1-3-pyridyl-1-butanol (NNAL). NNAL was quantified by liquid chromatography coupled to tandem mass spectrometry (LC-MS/MS). For total NNAL determination, urine samples were spiked with the NNAL – ^13^C_6_ internal standard, hydrolyzed with β-glucuronidase at 37°C for ≥24 h, extracted and purified on a purpose-designed molecularly imprinted polymer (MIP) solid-phase cartridge, and the analytes were eluted for LC-MS/MS analysis. The transitions m/z 210→180 (native) and m/z 216→186 (internal standard) were monitored. NNAL concentrations were calculated from the ratio of the integrated peak areas of the native and labeled ions against a standard calibration curve.

### Assessment of potential covariates

Potential covariates were selected based on previous epidemiological evidence and clinical relevance^18^ and categorized as follows: sex (male, female); age (20–39; 40–59; ≥60 years); race and ethnicity (Mexican American; other Hispanic; non-Hispanic White; non-Hispanic Black; other); education level (lower than high school; high school; higher than high school); family poverty-to-income ratio (<1.3; 1.3–3.4; >3.4); marital status (married, living with a partner/other); BMI (<25.0; 25.0–29.9; ≥30.0 kg/m^2^); alcohol consumption (yes, no); diabetes (yes, no); and hypertension (yes, no).

### Statistical analysis

Participant characteristics were first summarized for the full analytic sample. Urinary NNAL concentrations were categorized into three groups: Category 1, 0.0004–0.0017 ng/mL; Category 2, >0.0017 to 0.077925 ng/mL; and Category 3, >0.077925 ng/mL. Category 1 was used as the reference group in the logistic regression analyses to estimate the exposure–outcome relationship. The primary analyses incorporated the NHANES complex survey design, including the appropriate sampling weights, strata, and primary sampling units. Survey-weighted multivariable logistic regression models were used to estimate adjusted odds ratios (AORs) and corresponding 95% confidence intervals (CIs) for the association between urinary NNAL concentrations and OA. The primary analysis was restricted to participants with complete data on OA, urinary NNAL, and all included covariates. Three sequential models were constructed: Model 1 adjusted for age, sex, and race; Model 2 additionally adjusted for education level, family income, and marital status; Model 3 further adjusted for BMI, alcohol consumption, diabetes, and hypertension. Multicollinearity among covariates was assessed using variance inflation factors (VIFs), with VIF <5 considered indicative of no problematic multicollinearity. Three sensitivity analyses were conducted to evaluate the robustness of the primary survey-weighted complete-case analysis. First, the regression models were repeated without applying survey weights. Second, survey-weighted models were additionally adjusted for urinary creatinine to account for variation in urine dilution. Third, missing covariate values were imputed using single-value imputation, and the survey-weighted regression analyses were repeated in the expanded analytic sample.

Subsequently, subgroup analyses stratified by sex, age, and BMI were performed to examine whether the effect of NNAL on osteoarthritis differed across these strata. A two-sided p<0.05 was considered statistically significant. All statistical analyses were performed using R software (version 4.4.1; R Foundation for Statistical Computing, Vienna, Austria).

## RESULTS

### Participants characteristics

A total of 10848 participants met the eligibility criteria and were included in the final analysis (Figure 1). Among the 10848 participants, 2837 (26.2%) had OA and 8011 (73.8%) did not. Overall, 5031 participants (46.4%) were female; 3856 (35.5%) were aged 20–39 years, 3664 (33.8%) were aged 40–59 years, and 3328 (30.7%) were aged ≥60 years. More than half of the participants had an education level higher than high school (n=5677; 52.3%). Compared with participants without OA, those with OA had higher proportions of women (53.5% vs 43.9%), adults aged ≥60 years (57.8% vs 21.1%), hypertension (58.4% vs 25.8%), and diabetes (21.6% vs 8.2%) (all p<0.001). Urinary NNAL values also differed between participants with and without OA [0.20 (1.03) vs 0.15 (0.74) ng/mL; p=0.010] (Table 1).

**Table 1:** Participant characteristics according to osteoarthritis status among adults in a secondary cross-sectional analysis of data from NHANES, United States, 2007–2012 (N=10848)

*Characteristics*	*Total* *(N=10848)* *n (%)*	*No* *(N=801)* *n (%)*	*Yes* *(N=2837)* *n (%)*	*p*
**Gender**				<0.001
Female	5031 (46.38)	3513 (43.9)	1518 (53.5)	
Male	5817 (53.62)	4498 (56.1)	1319 (46.5)	
**Race**				<0.001
Mexican American	1581 (14.57)	1323 (16.5)	258 (9.09)	
Non-Hispanic Black	2266 (20.89)	1632 (20.4)	634 (22.3)	
Non-Hispanic White	5165 (47.61)	3553 (44.4)	1612 (56.8)	
Other Hispanic	1066 (9.83)	834 (10.4)	232 (8.18)	
Other	770 (7.10)	669 (8.35)	101 (3.56)	
**Age** (years)				<0.001
20–39	3856 (35.55)	3599 (44.9)	257 (9.06)	
40–59	3664 (33.78)	2725 (34.0)	939 (33.1)	
≥60	3328 (30.67)	1687 (21.1)	1641 (57.8)	
**Education level**				<0.001
Lower than high school	2702 (24.91)	1890 (23.6)	812 (28.6)	
High school	2469 (22.76)	1771 (22.1)	698 (24.6)	
Higher than high school	5677 (52.33)	4350 (54.3)	1327 (46.8)	
**Marital status**				0.040
No	4376 (40.34)	3185 (39.8)	1191 (42.0)	
Yes	6472 (59.66)	4826 (60.2)	1646 (58.0)	
**Family PIR**				<0.001
<1.3	3391 (31.26)	2463 (30.7)	928 (32.7)	
1.3–3.4	3993 (36.81)	2901 (36.2)	1092 (38.5)	
>3.4	3464 (31.93)	2647 (33.0)	817 (28.8)	
**Drinking alcohol**				<0.001
No	2329 (21.47%)	1420 (17.7)	909 (32.0)	
Yes	8519 (78.53)	6591 (82.3)	1928 (68.0)	
**BMI** (kg/m^2^)				<0.001
<25	3178 (29.30)	2551 (31.8)	627 (22.1)	
25–29.9	3640 (33.55)	2779 (34.7)	861 (30.3)	
≥30	4030 (37.15)	2681 (33.5)	1349 (47.6)	
**Hypertension**				<0.001
No	7128 (65.71)	5948 (74.2)	1180 (41.6)	
Yes	3720 (34.29)	2063 (25.8)	1657 (58.4)	
**Diabetes**				<0.001
No	9579 (88.3)	7355 (91.8)	2224 (78.4)	
Yes	1269 (11.7)	656 (8.19)	613 (21.6)	
**NNAL,** mean (SE)	0.16 (0.83)	0.15 (0.74)	0.20 (1.03)	0.010

BMI: body mass index. PIR: poverty income ratio. SE: standard error. All statistical tests were two-sided, and p<0.05 was considered statistically significant. NNAL: 4-(methylnitrosamino)-1-(3-pyridyl)-1-butanol. NHANES: National Health and Nutrition Examination Survey.

### Association between NNAL and osteoarthritis

Compared with participants in the lowest urinary NNAL category, participants in the two higher categories had the following adjusted estimates across the three regression models. In the primary survey-weighted analysis, compared with Category 1, the AORs for Category 2 were 1.52 (95% CI: 1.25–1.85; p<0.001) in Model 1, 1.37 (95% CI: 1.12–1.68; p=0.004) in Model 2, and 1.27 (95% CI: 1.02–1.56; p=0.038) in Model 3. The corresponding AORs for Category 3 were 1.76 (95% CI: 1.54–2.01; p<0.001), 1.50 (95% CI: 1.31–1.71; p<0.001), and 1.55 (95% CI: 1.36–1.76; p<0.001), respectively (Table 2). The primary findings were generally robust across the sensitivity analyses. In the unweighted analysis, the fully adjusted AORs were 1.10 (95% CI: 0.97–1.25; p=0.13) for Category 2 and 1.56 (95% CI: 1.37–1.78; p<0.01) for Category 3. After additional adjustment for urinary creatinine, the corresponding survey-weighted AORs were 1.27 (95% CI: 1.02–1.58; p=0.038) and 1.55 (95% CI: 1.34–1.78; p<0.001). After imputation of missing covariate data, the survey-weighted AORs were 1.24 (95% CI: 1.03–1.49; p=0.032) and 1.47 (95% CI: 1.33–1.61; p<0.001), respectively. The association for the highest NNAL category remained statistically significant across all sensitivity analyses.

**Table 2:** Association between urinary NNAL concentrations and osteoarthritis among adults in a secondary cross-sectional analysis of data from NHANES, United States, 2007–2012 (N=10848)

*NNAL^a^*	*Model 1^b^* *AOR (95% CI) p*	*Model 2* *AOR (95% CI) p*	*Model 3^d^* *AOR (95% CI) p*
**Weighted analysis^c^**			
Category 1 (ref.)	1	1	1
Category 2	1.52 (1.25–1.85) <0.001	1.37 (1.12–1.68) 0.004	1.27 (1.02–1.56) 0.038
Category 3	1.76 (1.54–2.01) <0.001	1.50 (1.31–1.71) <0.001	1.55 (1.36–1.76) <0.001
**Unweighted analysis**			
Category 1 (ref.)	1	1	1
Category 2	0.87 (0.78–0.96) <0.01	1.29 (1.15–1.46) <0.01	1.10 (0.97–1.25) 0.13
Category 3	1.11 (1.00–1.23) 0.05	1.82 (1.62–2.05) <0.01	1.56 (1.37–1.78) <0.01

a Category 1: 0.0004–0.0017 ng/mL; Category 2: >0.0017 to 0.077925 ng/mL; Category 3: >0.077925 ng/mL.

b Model 1: adjusted for age, sex, and race. Model 2: adjusted as for Model 1 plus education level, income level, and marital status. Model 3: adjusted as for Model 2 plus body mass index (BMI), alcohol consumption, diabetes, and hypertension.

c The primary analysis incorporated the appropriate NHANES sampling weights, strata, and primary sampling units. The unweighted analysis was performed as a sensitivity analysis. All statistical tests were two-sided, and p<0.05 was considered statistically significant.

d No evidence of problematic multicollinearity was observed; the maximum VIF was 3.97 for alcohol consumption, and all VIF values were <5. NNAL: 4-(methylnitrosamino)-1-(3-pyridyl)-1-butanol. NHANES: National Health and Nutrition Examination Survey.

### Subgroup analyses

In the survey-weighted subgroup analyses, the association between urinary NNAL and OA did not differ significantly by sex and BMI (p for interaction =0.231 and 0.127, respectively). Evidence of interaction was observed for age (p for interaction <0.001). Among adults younger than 60 years, the AOR for Category 3 was 1.69 (95% CI: 1.46–1.95; p<0.001), whereas among adults aged ≥60 years the corresponding AOR was 0.98 (95% CI: 0.82–1.17; p=0.823) (Table 3).

**Table 3:** Subgroup analyses of the association between urinary NNAL concentrations and osteoarthritis among adults in a secondary cross-sectional analysis of data from NHANES, United States, 2007–2012 (N=10848)

*Subgroup*	*n (%)*	*NNAL Category*	*AOR (95% CI)*	*p*	*p* *for interaction*
**Sex**					0.231
Men	5817 (53.62)	Category 1 (ref.)	1		
		Category 2	1.23 (1.06–1.42)	0.007	
		Category 3	1.32 (1.14–1.54)	<0.001	
Women	5031 (46.38)	Category 1 (ref.)	1	<0.001	
		Category 2	1.47 (1.26–1.73)		
		Category 3	1.53 (1.30–1.80)	<0.001	
**Age** (years)					<0.001
<60	7520 (69.32)	Category 1 (ref.)	1		
		Category 2	1.61 (1.40–1.85)	<0.001	
		Category 3	1.69 (1.46–1.95)	<0.001	
≥60	3328 (30.68)	Category 1 (ref.)	1		
		Category 2	0.91 (0.77–1.09)	0.304	
		Category 3	0.98 (0.82–1.17)	0.823	
**BMI** (kg/m^2^)					0.127
<25	3178 (29.30)	Category 1 (ref.)	1	<0.001	
		Category 2	1.67 (1.34–2.08)		
		Category 3	1.68 (1.34–2.09)	<0.001	
≥25	7670 (70.70)	Category 1 (ref.)	1		
		Category 2	1.28 (1.13–1.45)	<0.001	
		Category 3	1.33 (1.17–1.51)	<0.001	

AOR: adjusted odds ratio. BMI: body mass index. CI: confidence interval. NNAL: 4-(methylnitrosamino)-1-(3-pyridyl)-1-butanol. Association estimates were obtained using survey-weighted regression analyses incorporating the NHANES complex survey design. NNAL Category 1: 0.0004–0.0017 ng/mL; Category 2: >0.0017 to 0.077925 ng/mL; Category 3: >0.077925 ng/mL. All statistical tests were two-sided, and p<0.05 was considered statistically significant. NHANES: National Health and Nutrition Examination Survey.

## DISCUSSION

In this secondary cross-sectional analysis of a nationally representative sample of 10848 US adults, higher urinary NNAL concentrations were associated with greater odds of OA. This association was particularly pronounced among individuals aged <60 years. These findings suggest that higher tobacco exposure, as reflected by urinary NNAL concentrations, may be associated with a greater likelihood of OA in selected demographic groups.

These findings provide essential evidence that enhances the evolving understanding of the factors associated with smoking and joint health, addressing previous inconsistencies in the literature. While some earlier studies indicated no significant association between smoking and OA^19^, this discrepancy may be attributable to differences in study design and population. The Chingford study enrolled only 1003 women aged 45–64 years from a single UK community and statistically adjusted only for age and body mass index, whereas our analysis draws on a nationally representative sample of 10848 US adults spanning a broader age range and both sexes, with adjustment for a considerably larger set of covariates, providing greater statistical power, generalizability, and confounder control. That earlier study also relied on radiographically confirmed OA at specific joint sites and self-reported ever/never smoking status, whereas the present study used self-reported physician-diagnosed OA and urinary NNAL as the exposure measure, differences that may further contribute to the divergent findings. Our utilization of NNAL, a validated, objective biomarker of TSNA exposure, mitigates the biases associated with self-reported smoking status. MRI-based studies have shown that current smokers had higher cartilage T2 values than non-smokers and greater cartilage loss in weight-bearing knee compartments, providing additional evidence of an association between smoking and structural cartilage damage^20,21^.

Urinary NNAL provides a quantitative biomarker of exposure to NNK, a tobacco-specific nitrosamine with systemic toxic effects^22^. NNK, rather than urinary NNAL, may undergo systemic distribution and tissue retention, whereas NNAL is measured in urine as a metabolite and biomarker of NNK exposure^23^. This finding complements preclinical evidence indicating that nicotine, a principal component of tobacco, disrupts chondrogenesis and impairs cartilage repair^24^. Given the cross-sectional design, however, this exposure specificity does not establish causality.

In subgroup analyses, significant associations were observed among men, women, and across BMI subgroups, whereas significant heterogeneity in the association was observed by age. The differing estimates across age groups may partly reflect age-related changes in cartilage physiology. Articular chondrocytes exhibit age-related declines in proliferative and synthetic capacity, while aging cartilage is characterized by impaired anabolic responses and reduced maintenance of tissue homeostasis^25^. However, whether these mechanisms explain the observed age-stratified heterogeneity cannot be determined from the present cross-sectional analysis. In contrast, aging joints are often predominantly affected by age-related degenerative processes. This observation is consistent with findings that indicate smoking exacerbates cartilage loss in younger adults with preexisting OA^20^, suggesting that tobacco exposure may intensify early joint damage prior to the onset of irreversible age-related degeneration.

Several biological pathways may elucidate the observed association. Firstly, smoking induces a chronic inflammatory state, characterized by the release of pro-inflammatory cytokines such as TNF-α and IL-6, which are known to degrade the cartilage matrix and facilitate the progression of OA. Secondly, preclinical studies indicate that nicotine and TSNAs inhibit the chondrogenic differentiation of mesenchymal stem cells and downregulate the expression of IGF-1 in chondrocytes, thereby impairing cartilage repair processes^24^. Thirdly, tobacco exposure modifies bone turnover markers, including serum bone alkaline phosphatase and urinary N-telopeptide, which disrupts the bone-cartilage crosstalk essential for maintaining joint homeostasis^26^. Collectively, these mechanisms provide biological plausibility for the observed association but do not establish a causal relationship between tobacco exposure and OA.

### Strengths and limitations

The strengths of this study include the use of a nationally representative sample, a large analytic sample size, and urinary NNAL as an objective biomarker of tobacco exposure^27^. The use of urinary NNAL may reduce recall bias and exposure misclassification associated with self-reported smoking behavior^28^. Despite these strengths, several limitations should be considered. First, the cross-sectional design precluded assessment of temporality or causality, and residual confounding by unmeasured or incompletely measured factors, including physical activity and previous joint injury, could not be excluded. Second, self-reported physician-diagnosed OA and questionnaire-based covariates may have been affected by recall or reporting bias. Third, participants with missing outcome, exposure, or covariate data were excluded. If these data were not missing completely at random, the complete-case analysis may have introduced selection bias. Fourth, information on OA severity and specific joint involvement was unavailable, limiting the assessment of heterogeneity by disease phenotype. Fifth, a single urinary NNAL measurement may not fully represent long-term tobacco exposure^29^. Finally, although NHANES is nationally representative, the findings are primarily generalizable to the non-institutionalized US adult population and may not apply directly to institutionalized individuals or populations in other countries.

## CONCLUSIONS

In this secondary cross-sectional analysis of NHANES 2007–2012, higher urinary NNAL concentrations were associated with greater odds of OA. In stratified analyses, statistically significant associations were observed among adults younger than 60 years. Because the cross-sectional design precludes assessment of temporality or causality, longitudinal studies are needed to confirm these findings.

## Data Availability

The datasets analyzed in this study are publicly available from the NHANES, National Center for Health Statistics, Centers for Disease Control and Prevention. NHANES public-use datasets and accompanying documentation can be accessed through the official NHANES website (https://wwwn.cdc.gov/nchs/nhanes/).
